# Dataset of statements on policy integration of selected intergovernmental organizations

**DOI:** 10.1016/j.dib.2018.02.064

**Published:** 2018-02-28

**Authors:** Jale Tosun, B. Guy Peters

**Affiliations:** a Institute of Political Science, Heidelberg University, Bergheimer Straße 58, 69115 Heidelberg, Germany; b Heidelberg Center for the Environment, Im Neuenheimer Feld 229, 69120 Heidelberg, Germany; c Department of Political Science, University of Pittsburgh, 4812 Posvar Hall, Pittsburgh, Pennsylvania 15260, United States

## Abstract

This article describes data for 78 intergovernmental organizations (IGOs) working on topics related to energy governance, environmental protection, and the economy. The number of IGOs covered also includes organizations active in other sectors. The point of departure for data construction was the Correlates of War dataset, from which we selected this sample of IGOs. We updated and expanded the empirical information on the IGOs selected by manual coding. Most importantly, we collected the primary law texts of the individual IGOs in order to code whether they commit themselves to environmental policy integration (EPI), climate policy integration (CPI) and/or energy policy integration (EnPI).

**Specifications Table**

**Table:** 

Subject area	*Political Science*
More specific subject area	*Statements of intergovernmental organizations on policy integration*
Type of data	*Table*
How data was acquired	*Updating and expansion of an existing dataset by means of manual coding*
Data format	*Raw and analyzed*
Experimental factors	*Not applicable*
Experimental features	*Not applicable*
Data source location	*Online*
Data accessibility	The data are available within this article; additional data are available at: http://dx.doi.org/10.17632/kk9gvbd84k.1#file-e5723935-b1bc-4759-8e92-f4a5e51bbddd

**Value of the data**

• The data offer insights in how IGOs incorporate policy integration in their primary law texts.
• The data are unique for they combine information on EPI, CPI and EnPI as different conceptualizations of policy integration.
• The data build on innovative conceptualizations of policy integration as ‘coordination’ and ‘harmonization’ as well as differentiate between ‘internal’ and ‘external’ forms of policy integration.
• The coding of EPI, CPI and EnPI has the potential to guide future research in comparative policy analysis.

## 1 Data

We are sharing three types of data with the readers. First, an overview of the 78 IGOs for which we gathered new data (Table 1). Second, raw data on the relevant statements in the 78 IGOs’ primary law texts and information on how we coded the statements concerned (Table 2). The first two types of data are only available within this article. The third data component comprises the coded information on EPI, CPI and EnPI together with the measurement of the explanatory variables used by Tosun and Peters (2018) for testing their hypotheses (Table 3) [1]. Lastly, we offer robustness checks of the analyses run by Tosun and Peters in Table 4, Table 5.

**Table 1:** Overview of IGOs included in the dataset.

**ID**	**CoW ID**	**IGO**	**Short**	**Founded**^**1**^	**Text year**	**Primary law**	**Sector**^**2**^
1	2710	International Energy Agency	IEA	1974	2014	Agreement (amended)	Energy
2		International Energy Forum^3^	IEF	1991	2011	Charter (amended)	Energy
3	3410	Latin American Energy Organization	OLADE	1973	1973	Agreement	Energy
4		Energy Community Treaty^4^	ECT	2005	2017	Treaty (amended)	Energy
5	2370	International Atomic Energy Agency	IAEA	1957	1989	Statute (amended)	Energy
6	3840	Organization of Petroleum Exporting Countries	OPEC	1960	2012	Statute (amended)	Energy
7	3800	Organization of Arab Petroleum Exporting Countries	OAPEC	1968	1968	Statute	Energy
8		International Renewable Energy Agency^3^	IRENA	2009	2009	Statute	Energy
9	310	Amazon Cooperation Treaty	ACT	1978	1998	Treaty/Protocol (amended)	Environment
10	530	Arctic Council	AC	1996	1996	Declaration	Environment
11	2060	Asia Pacific Fishery Commission	APFIC	1948	1996	Agreement (amended)	Environment
12	300	Agreement for Cooperation in Dealing with Pollution of the North Sea	Bonn Agreement	1983	2001	Agreement (amended)	Environment
13	1130	Plant Protection Committee of the Southern Cone	COSAVE	1989	1989	Convention	Environment
14	1450	Desert Locust Control Organization for East Africa	DLCO-EA	1962	1965	Convention (amended)	Environment
15	1560	European and Mediterranean Plant Protection Organization	EPPO	1951	1999	Convention (amended)	Environment
16	1940	Group of Temperate Southern Hemisphere Countries on the Environment	Valdivia Group	1995	1995	Declaration	Environment
17	1960	Group on Earth Observations	GEO	2005	2004	Framework/Resolution	Environment
					2005		
18	2300	Intergovernmental Organization for Marketing Information and Technical Advisory Services for Fishery Products in the Asia and Pacific Region	INFOFISH	1985	1985	Agreement	Environment
19	2290	Intergovernmental Oceanographic Commission of UNESCO	IOC-UNESCO	1960	1999	Statute (amended)	Environment
20	2450	International Commission for the Protection of the Rhine	ICPR	1950	1999	Convention (amended)	Environment
21	2570	International Commission for the Northwest Atlantic Fisheries	ICNAF	1949	1974	Convention (amended)	Environment
22	2770	International Fund for Saving the Aral Sea	IFAS	1993	1999	Agreement (amended)	Environment
23	2950	International Oil Pollution Compensation Funds	IOPC Funds	1971	2003	Convention (amended)	Environment
24	3020	International Treaty on Plant Genetic Resources for Food and Agriculture	IT PGRFA	2001	2001	Treaty	Environment
25	3060	International Regional Organization Of Animal And Plant Health	OIRSA	1953	1987	Convention (amended)	Environment
26	3250	International Whaling Commission	IWC	1946	1946	Convention	Environment
27	3420	Latin American Fisheries Development Organization	OLDEPESCA	1982	1982	Agreement	Environment
28	1150	Lake Chad Basin Commission	LCBC	1964	1990	Convention/Statute (amended)	Environment
29	3680	North American Plant Protection Organization	NAPPO	1976	2004	Constitution (amended)	Environment
30	2920	North Pacific Anadromous Fish Commission	NPAFC	1952	1992	Convention (amended)	Environment
31	3730	North-East Atlantic Fisheries Commission	NEAFC	1978	1978	Convention	Environment
32	3930	Permanent Commission of the Conference on the Exploitation and Conservation of the Maritime Resources of the South Pacific	CPPS	1952	2012	Statute	Environment
33	3970	Permanent Interstate Committee for Drought Control in the Sahel	CILSS	1973	1994	Convention (amended)	Environment
34	4160	South Asia Cooperative Environment Program	SACEP	1981	1981	Declaration	Environment
35	1356	Central Africa Forest Commission	COMIFAC	1999	2005	Treaty (amended)	Environment
36	4360	International Union for Protection of New Varieties of Plants	UPOV	1961	1991	Convention (amended)	Environment
37		International Bank for Reconstruction and Development (World Bank)	IBRD	1944	2012	Agreement (amended)	Economy: Bank/Fund
38	2750	International Finance Corporation (World Bank)	IFC	1956	2012	Agreement (amended)	Economy: Bank/Fund
39	3540	Multilateral Investment Guarantee Agency (World Bank)	MIGA	1988	2010	Convention (amended)	Economy: Bank/Fund
40	2160	Inter‐American Development Bank	IADB	1959	1996	Agreement (amended)	Economy: Bank/Fund
41	70	African Development Bank	AfDB	1964	2016	Agreement (amended)	Economy: Bank/Fund
42	580	Asian Development Bank	ADB	1966	1966	Agreement	Economy: Bank/Fund
43	1570	European Bank for Reconstruction and Development	EBRD	1991	2013	Agreement (amended)	Economy: Bank/Fund
44	3320	Islamic Development Bank	IsDB	1974	1974	Agreement	Economy: Bank/Fund
45	3630	Nordic Development Fund	NDF	1989	2011	Legal Framework (amended)	Economy: Bank/Fund
46	3190	International Tropical Timber Organization	ITTO	1985	2006	Agreement (amended)	Economy: Commodity
47	2520	International Cocoa Organization	ICCO	1973	2010	Agreement (amended)	Economy: Commodity
48	2530	International Coffee Organization	ICO	1963	2007	Agreement (amended)	Economy: Commodity
49	2660	International Copper Study Group	ICSG	1992	2015	Terms of Reference (amended)	Economy: Commodity
50	2910	International Nickel Study Group	INSG	1990	2015	Terms of Reference (amended)	Economy: Commodity
51	3090	International Rubber Study Group	IRSG	1944	2011	Constitution (amended)	Economy: Commodity
52	3260	International Grains Council	IGC	1949	1995	Convention (amended)	Economy: Commodity
53	1920	Group of Latin American and Caribbean Sugar Exporting Countries	GEPLACEA	1977	1977	Statute	Economy: Commodity
54	2960	International Olive Council	IOC	1959	2015	Agreement (amended)	Economy: Commodity
55	4420	United Nations Industrial Development Organization	UNIDO	1966	1979	Constitution (amended)	Economy: Other
56	2760	International Fund for Agricultural Development	IFAD	1977	1977	Agreement	Economy: Other
57	4580	World Trade Organization	WTO	1995	1995	Agreement	Economy: Other
58	1520	Economic Community of West African States	ECOWAS	1975	1993	Treaty (amended)	Economy: Other
59	3810	Black Sea Economic Cooperation	BSEC	1992	1998	Charter	Economy: Other
60	4260	Southern Common Market	MERCOSUR	1991	1991	Treaty	Economy: Other
61	3670	North American Free Trade Agreement	NAFTA	1994	1994	Agreement	Economy: Other
62	3750	Organization for Economic Co-operation and Development	OECD	1960	1960	Convention	Economy: Other
63	330	Andean Community	CAN	1969	1969	Agreement	Economy: Other
64	4550	World Health Organization	WHO	1946	2005	Constitution (amended)	Health
65	4500	West African Health Organization	WAHO	1987	1987	Protocol	Health
66	2070	Institute of Nutrition of Central America and Panama	INCAP	1949	1998	Convention (amended)	Health
67	1840	UN Food and Agriculture Organization	FAO	1945	1945	Constitution	Multi-issue
68	1230	Commonwealth of Independent States	CIS	1993	1993	Agreement	Multi-issue
69	4200	Pacific Islands Forum	PIF	1971	2005	Agreement (amended)	Multi-issue
70	4530	World Meteorological Organization	WMO	1950	2015	Convention (amended)	Other
71	4570	World Tourism Organization	UNWTO	1970	2011	Statute (amended)	Other
72	4540	Working Community of the Danube Countries	ARGE	1990	1990	Declaration	Other
73	1720	European Organization for Nuclear Research	CERN	1954	1971	Convention (amended)	Science/Education
74	4220	Southeast Asian Ministers of Education Organization	SEAMEO	1965	2013	Charter (amended)	Science/Education
75	4410	United Nations Educational, Scientific, and Cultural Organization	UNESCO	1945	1945	Constitution	Science/Education
76	2500	International Civil Aviation Organization	ICAO	1919	2006	Convention (amended)	Transport
77	2860	International Maritime Organization	IMO	1948	1993	Convention (amended)	Transport
78	4010	Port Management Association of Eastern and Southern Africa	PMAESA	1973	2004	Constitution (amended)	Transport

Notes: Information on founding date according to the IGOs’ websites^1^; sector categorizations are taken from Blake and Payton [3]^2^; an exception is the coding category ‘energy’ and the sector assigned to the organization ICAO. IGOs are taken from the CoW dataset [2]; alternative sources used for more recent energy-related IGOs are Van de Graaf and Colgan [4]^3^ and Blake and Payton [3]^4^. To faciliate the comparison of these data with the CoW dataset, the table also reports the identification numbers used in the corresponding CoW codebook [2].

**Table 2:** Relevant statements on policy integration in the (amended) primary law texts.

**ID**	**Short name (Relevant articles)**	**Extracts of the text used for coding purposes (*****Clarification on how the statement was coded with regard to internal/external policy integration and coordination/harmonization*****)**
1.	IEA	Determined to reduce their dependence on imported oil by undertaking long-term co-operative efforts on conservation of energy, on accelerated development of alternative sources of energy, on research and development in the energy field and on uranium enrichment (*Internal: coordination*)
2.	IEF	The fundamental aims of the Forum are:
		a. fostering greater mutual understanding and awareness of common energy interests among its Members;
		b. promoting a better understanding of the benefits of stable and transparent energy markets for the health of the world economy, the security of energy supply and demand, and the expansion of global trade and investment in energy resources and technology;
		c. identifying and promoting principles and guidelines that enhance energy market transparency, stability and sustainability;
		d. narrowing the differences among energy producing, consuming and transit Member States on global energy issues and promoting a fuller understanding of their interdependency and the benefits to be gained from cooperation through dialogue among them, as well as between them and energy related industries;
		e. promoting the study and exchange of views on the inter-relationships among energy, technology, environmental issues, economic growth and development;
		f. building confidence and trust through improved information sharing among States; and
		g. facilitating the collection, compilation and dissemination of data, information and analyses that contribute to greater market transparency, stability and sustainability. (*Internal: harmonization; external: coordination*)
3.	OLADE	To promote among the Member States the adoption of effective measures to prevent environmental pollution due to the exploitation, transportation, storage, and utilization of the energy resources of the Region and to recommend the measures deemed necessary to prevent environmental pollution caused by the exploitation, transportation, storage, and utilization of the energy resources within the Region, in areas not under the jurisdiction of the Member States (*Internal: harmonization; external: coordination*)
4.	ECT	1. The task of the Energy Community shall be to organise the relations between the Parties and create a legal and economic framework in relation to Network Energy, as defined in paragraph 2, in order to:
		(a) create a stable regulatory and market framework capable of attracting investment in gas networks, power generation, and transmission and distribution networks, so that all Parties have access to the stable and continuous energy supply that is essential for economic development and social stability,
		(b) create a single regulatory space for trade in Network Energy that is necessary to match the geographic extent of the concerned product markets,
		(c) enhance the security of supply of the single regulatory space by providing a stable investment climate in which connections to Caspian, North African and Middle East gas reserves can be developed, and indigenous sources of energy such as natural gas, coal and hydropower can be exploited,
		(d) improve the environmental situation in relation to Network Energy and related energy efficiency, foster the use of renewable energy, and set out the conditions for energy trade in the single regulatory space,
		(e) develop Network Energy market competition on a broader geographic scale and exploit economies of scale (*Internal: harmonization; external: harmonization*)
5.	IAEA	No relevant statement
6.	OPEC	No relevant statement
7.	OAPEC	No relevant statement
8.	IRENA	The Agency shall promote the widespread and increased adoption and the sustainable use of all forms of renewable energy, taking into account:
		a.) national and domestic priorities and benefits derived from a combined approach of renewable energy and energy efficiency measures, and
		b.) the contribution of renewable energy to environmental preservation, through limiting pressure on natural resources and reducing deforestation, particularly tropical deforestation, desertification and biodiversity loss; to climate protection; to economic growth and social cohesion including poverty alleviation and sustainable development; to access to and security of energy supply; to regional development and to inter-generational responsibility. *(Internal: harmonization; external: harmonization*)
9.	ACT	No relevant statement
10.	AC	No relevant statement
11.	APFIC	No relevant statement
12.	Bonn Agreement	No relevant statement
13.	COSAVE (Article 1; in Spanish only)	Los Países Miembros constituyen el Comité Regional de Sanidad Vegetal COSAVE, con el objetivo principal de coordinar e incrementar la capacidad regional de prevenir, disminuir y evitar los impactos y riesgos de los problemas que afectan a la producción y comercialización de los productos agrícolas y forestales de la región, tomando en cuenta la situación fitosantiaria alcanzada, el desarrollo económico sostenido, salud humana y la protección del medio ambiente. (*Internal: harmonization*)
14.	DLCO-EA	No relevant statement
15.	EPPO	There shall be established a European and Mediterranean Plant Protection Organization (hereinafter referred to as the Organization), as a recognized regional plant protection organization under the International Plant Protection Convention, established by the Food and Agriculture Organization of the United Nations (FAO)1. The aims of the Organization are:
		a. to support the Member Governments in their aim of assuring plant health, while preserving human and animal health and the environment;
		b. to pursue and develop, by cooperation between the Member Governments, the protection of plants and plant products against pests and the prevention of their international spread and especially their introduction into endangered areas;
		c. to develop internationally harmonized phytosanitary and other official plant protection measures and, as appropriate, to elaborate standards to that effect;
		d. to present the collective views of the Member Governments, as appropriate, to FAO, WTO, other regional plant protection organizations and any other bodies with related responsibilities. (*Internal: harmonization*)
16.	Valdivia Group	3. In order to commence the work of the Group, representatives agreed to establish initially five Working Groups coordinated by the countries indicated:
		a. Climate Change (Argentina)
		b. Biological Diversity (Australia)
		c. Forests (Chile)
		d. Ozone (New Zealand)
		e. Desertification (South Africa)
		(*external: harmonization*)
17.	GEO	Observing and understanding the Earth system more completely and comprehensively will expand worldwide capacity and means to achieve sustainable development and will yield advances in many specific areas of socio-economic benefit, including:
		• Reducing loss of life and property from natural and human-induced disasters; • Understanding environmental factors affecting human health and well being; • Improving management of energy resources; • Understanding, assessing, predicting, mitigating, and adapting to climate variability and change; • Improving water resource management through better understanding of the water cycle; • Improving weather information, forecasting, and warning; • Improving the management and protection of terrestrial, coastal, and marine ecosystems; • Supporting sustainable agriculture and combating desertification; • Understanding, monitoring, and conserving biodiversity. (*internal: harmonization; external: harmonization*)
18.	INFOFISH	No relevant statement
19.	IOC-UNESCO	No relevant statement
20.	ICPR	No relevant statement
21.	ICNAF	No relevant statement
22.	IFAS	No relevant statement
23.	IOPC Funds	No relevant statement
24.	IT PGRFA	No relevant statement
25.	OIRSA	No relevant statement
26.	IWC	No relevant statement
27.	OLDEPESCA	That, it is necessary to encourage the correct use and protection of fishery resources within its maritime jurisdiction zones of each State whilst preserving the marine and freshwater environment and applying rational conservation policies for the same which entails mutual cooperation and the development of joint programs; (*internal: harmonization*)
28.	LCBC	No relevant statement
29.	NAPPO	No relevant statement
30.	NPAFC	No relevant statement
31.	NEAFC	No relevant statement
32.	CPPS	No relevant statement
33.	CILSS	No relevant statement
34.	SACEP	• Environmental Impact Assessment and Cost *I* Benefit Analysis; Environment and Development. • Environment Quality Standards • Technology for the Development of Renewable and Reusable Resources. • Environment Legislation. • Conservation of Montane Eco-Systems and Watersheds. • Social Forestry. • Regional Co-operation in Wild Life and Genetic Resources Conservation. • Conservation of orals, Mangroves, Deltas, Coastal Areas and • Island Eco-Systems. • Tourism & Environment. • Desertification. • Regional Seas Programme. • Energy & Environment. • Education and Training. • Training in Wild Life Management.
		(*external: harmonization*)
35.	COMIFAC (in French)	Mettre en place des mesures destinées à conciliar les actions en faveur de la conservation et de la gestion durable des écosystèmes forestiers aves les politiques de développement dans d’autres secteurs, notamment le reboisement, les transport et l’agriculture; (*internal: reforestation*)
36.	UPOV	No relevant statement
37.	IBRD	No relevant statement
38.	IFC	No relevant statement
39.	MIGA	No relevant statement
40.	IADB	No relevant statement
41.	AfDB	No relevant statement
42.	ADB	No relevant statement
43.	EBRD	vii) to promote in the full range of its activities environmentally sound and sustainable development (*external: harmonization*)
44.	IsDB	No relevant statement
45.	NDF	No relevant statement
46.	ITTO	(m) Encouraging members to develop national policies aimed at sustainable utilization and conservation of timber producing forests, and maintaining ecological balance, in the context of the tropical timber trade; (*external: harmonization*)
47.	ICCO	(e) To promote a sustainable cocoa economy in economic, social and environmental terms; (*external: harmonization*)
48.	ICO	(3) encouraging Members to develop a sustainable coffee sector in economic, social and environmental terms; (*external: harmonization*)
49	ICSG	No relevant statement
50.	INSG	No relevant statement
51.	IRSG	No relevant statement
52.	IGC	No relevant statement
53	GEPLACEA	No relevant statement
54.	IOC	To study the interaction between olive growing and the environment, particularly with a view to promoting environmental conservation and sustainable production, and to ensure the integrated and sustainable development of the sector; (*external: harmonization*)
55.	UNIDO	(I) Advise on and assist, in close co-operation with the appropriate bodies of the. United Nations, specialized agencies and the International Atomic Energy, the developing countries in the exploitation, conservation and local transformation of their natural resources for the purpose of furthering the industrialization of developing countries. (*external: coordination*)
56.	IFAD	No relevant statement
57.	WTO	Recognizing that their relations in the field of trade and economic endeavour should be conducted with a view to raising standards of living, ensuring full employment and a large and steadily growing volume of real income and effective demand, and expanding the production of and trade in goods and services, while allowing for the optimal use of the world's resources in accordance with the objective of sustainable development, seeking both to protect and preserve the environment and to enhance the means for doing so in a manner consistent with their respective needs and concerns at different levels of economic development, (*external: harmonization*)
58.	ECOWAS (Article 3)	1. The aims of the Community are to promote co-operation and integration, leading to the establishment of an economy union in West Africa in order to raise the living standards of its peoples, and to maintain and enhance economic stability, foster relations-among Member States and contribute to the progress and development of the African Continent.
		2. In order to achieve the aims set out in the paragraph above, and in accordance with the relevant provisions of this Treaty, the Community shall, by stages, ensure;
		a) the harmonization and co-ordination of national policies and the promotion of integration programmes, projects and activities, particularly in food, agriculture and natural resources, industry, transport and communications, energy, trade, money and finance, taxation, economic reform policies, human resources, education, information, culture, science, technology, services, health, tourism, legal matters;
		b) the harmonization and co-ordination of policies for the protection of the environment; (*external: harmonization*)
59.	BSEC	In accordance with the agreed principles and with the aim of utilizing more effectively their human, natural and other resources for attaining a sustained growth of their national economies and the social well-being of their peoples, the Member States shall cooperate in the following areas: trade and economic development; banking and finance; communications; energy; transport; agriculture and agro-industry; health care and pharmaceutics; environmental protection; tourism; science and technology; exchange of statistical data and economic information; collaboration between customs and other border authorities; human contacts; combating organized crime, illicit trafficking of drugs, weapons and radioactive materials, all acts of terrorism and illegal migration, or in any other related area, following a decision of the Council. (*external: harmonization*)
60.	MERCOSUR	CONSIDERING that the expansion of their domestic markets, through integration, is a vital prerequisite for accelerating their processes of economic development with social justice,
		BELIEVING that this objective must be achieved by making optimum use of available resources, preserving the environment, improving physical links, coordinating macroeconomic policies and ensuring complementarily between the different sectors of the economy, based on the principles of gradualism, flexibility and balance, (*external: harmonization*)
61.	NAFTA (Chapters 1 and 6)	UNDERTAKE each of the preceding in a manner consistent with environmental protection and conservation;
		PRESERVE their flexibility to safeguard the public welfare;
		PROMOTE sustainable development;
		STRENGTHEN the development and enforcement of environmental laws and regulations;
		2. The Parties recognize that it is desirable to strengthen the important role that trade in energy and basic petrochemical goods plays in the free trade area and to enhance this role through sustained and gradual liberalization.
		(*external: harmonization*)
62.	OECD	No relevant statement
63.	CAN (in Spanish)	Complementariamente a los mecanismos antes enunciados, se adelantarán, en forma concertada, los siguientes programas y acciones de cooperación económica y social:
		a) Programas orientados a impulsar el desarrollo científico y tecnológico; b) Acciones en el campo de la integración fronteriza; c) Programas en el área del turismo; d) Acciones para el aprovechamiento y conservación de los recursos naturales y del medio ambiente; e) Programas de desarrollo social; y, f) Acciones en el campo de la comunicación social. (*external: harmonization*)
64.	WHO	In order to achieve its objective, the functions of the Organization shall be:
		(i) to promote, in co-operation with other specialized agencies where necessary, the improvement of nutrition, housing, sanitation, recreation, economic or working conditions and other aspects of environmental hygiene; (*external: coordination*)
65.	WAHO	No relevant statement
66.	INCAP	No relevant statement
67.	FAO	2. The Organization shall promote and, where appropriate, shall recommend national and international action with respect to:
		(a) scientific, technological, social and economic research relating to nutrition, food and agriculture;
		(b) the improvement of education and administration relating to nutrition, food and agriculture, and the spread of public knowledge of nutritional and agricultural science and practice;
		(c) the conservation of natural resources and the adoption of improved methods of agricultural production;
		(d) the improvement of the processing, marketing and distribution of food and agricultural products;
		(e) the adoption of policies for the provision of adequate agricultural credit, national and international;
		(f) the adoption of international policies with respect to agricultural commodity arrangements. (*external: harmonization*)
68.	CIS	Bodies of Branch Cooperation
		• Anti –Terrorist Center • Interstate Bank • Interstate Statistical Committee • Interstate Council on Standardization Metrology and Certification • Interstate Council on Emergency Situation of Natural and Anthropogenic Character • Interstate Council on Antimonopoly Policy • Coordinating council of the states-participants of the CIS on Informatization under the Regional Commonwealth in the Field of Communications • Electric Energy Council • Interstate Council on Aviation and Air Space Use • Council of the Heads of Statistical Services of the States-Participants of the Commonwealth • Council of the Heads of Customs Services of the States-Participants of the Commonwealth • and other (*external: harmonization*)
69.	PIF	Determined to work in partnership with each other and with others beyond our region to achieve our shared goals of economic growth, sustainable development, good governance and security; (*external: harmonization*)
70.	WMO	Considering further the need for close cooperation with other international organizations also working in the areas of hydrology, climate and environment (*external: harmonization*)
71.	UNWTO	No relevant statement
72.	ARGE (Article 2; in German)	Aufgabe der Arbeitsgemeinschaft ist die gemeinsame, informative und fachliche Behandlung und Koordinierung von Fragen, welche in Interesse ihrer Mitglieder liegen. Insbesondere sollen Fragen der Wirtschaft, der Raumordnung, des Verkehrs, des Natur- und Umweltschutzes, des Fremdenverkehrs und der kulturellen und wissenschaftlichen Kontakte behandelt werden. Bestehende bilaterale und multilaterale Kontakte zwischen den Mitgliedern werden durch die Tätigkeit der Arbeitsgemeinschaft nicht beeinträchtigt; sie können im Rahmen der Möglichkeiten unterstützt werden. (*external: harmonization*)
73.	CERN	No relevant statement
74.	SEAMEO	No relevant statement
75.	UNESCO	No relevant statement
76.	ICAO	No relevant statement
77.	IMO (Article 1)	(a) To provide machinery for co-operation among Governments in the field of governmental regulation and practices relating to technical matters of all kinds affecting shipping engaged in international trade, and to encourage the general adoption of the highest practicable standards in matters concerning maritime safety, efficiency of navigation and prevention and control of marine pollution from ships; and to deal with administrative and legal matters related to the purposes set out in this Article. (*external: coordination*)
78.	PMAESA	No relevant statement

**Table 3:** Summary statistics.

Variable	Obs	Mean	Std. Dev.	Min	Max
EPI	50	0.44	0.50	0	1
Multi-region	78	0.47	0.50	0	1
Europe	78	0.12	0.32	0	1
France	78	0.54	0.50	0	1
EU Commission	78	0.22	0.42	0	1
Energy	78	0.06	0.25	0	1
Bank	78	0.10	0.31	0	1
Commodity	78	0.12	0.32	0	1
Economy general	78	0.12	0.32	0	1
Multi-issue	78	0.06	0.25	0	1
Other	78	0.18	0.39	0	1
Membership size	78	5.41	2.92	1	10
United Nations	78	0.18	0.39	0	1
Post 1987	78	0.74	0.44	0	1
Text age	78	5.37	2.92	1	10

**Table 4:** Determinants of IGO commitment to EPI (robustness check; United Kingdom replaced by France).

	Model 1		Model 2		Model 3		Model 4	
	β	S.E.	β	S.E.	β	S.E.	β	S.E.
Multi-region	−3.584	(1.769)^**^	−2.950	(1.486)^**^				
Europe					3.540	(1.753)^**^	2.584	(1.446)^*^
France	0.996	(1.251)	1.648	(1.173)				
EU Commission	2.171	(1.062)^**^	1.859	(0.983)^*^	1.173	(0.906)		
Energy	1.827	(1.404)	2.089	(1.425)	1.464	(1.397)	1.494	(1.240)
Bank	−2.810	(2.005)	−1.481	(1.721)	−2.367	(1.871)	−1.977	(1.607)
Commodity	1.986	(1.245)	2.510	(1.458)^*^	1.166	(1.082)	0.960	(0.968)
Economy general	4.856	(1.811)^***^	3.967	(1.473)^***^	4.490	(1.609)^***^	3.346	(1.252)^***^
Multi-issue	2.351	(1.313)^*^	2.530	(1.367)^*^	2.832	(1.403)^**^	2.240	(1.273)^*^
Other	– base level –							
Membership size	0.469	(0.291)			0.236	(0.172)		
United Nations			1.895	(1.273)				
Post 1987	2.654	(1.267)^**^	2.199	(1.110)^**^	2.335	(1.206)^*^	1.869	(0.992)^*^
Constant	−5.612	(2.276)^**^	−3.624	(1.463)^**^	−5.416	(2.257)^**^	−2.892	(1.132)^**^
N	50		50		50		50	
AIC	67.655		68.175		66.009		66.407	

Notes: Dependent variable = normative commitment to EPI (binary variable); β = logit coefficient; S.E. = standard error; AIC = Akaike Information Criterion; Significance levels: ^*^ p< 0.10, ^**^ p < 0.05; ^***^ p < 0.01.

**Table 5:** Determinants of IGO commitment to EPI (robustness check; Post 1987 replaced by Text age).

	Model 1		Model 2		Model 3		Model 4	
	β	S.E.	β	S.E.	β	S.E.	β	S.E.
Multi-region	−3.341	(1.725)⁎	−3.810	(1.811)⁎⁎				
Europe					3.377	(1.717)⁎⁎	2.686	(1.463)⁎
United Kingdom	0.897	(1.206)	1.710	(1.187)				
EU Commission	2.246	(1.089)⁎⁎	2.163	(1.053)⁎⁎	1.224	(0.922)		
Energy	1.963	(1.464)	2.407	(1.464)	1.807	(1.445)	1.783	(1.307)
Bank	−3.290	(2.194)	−2.640	(2.131)	−2.715	(1.997)	−2.295	(1.706)
Commodity	1.674	(1.178)	2.773	(1.489)⁎	0.988	(1.061)	0.903	(0.976)
Economy general	4.319	(1.610)⁎⁎⁎	4.031	(1.444)⁎⁎⁎	4.254	(1.519)⁎⁎⁎	3.477	(1.242)⁎⁎⁎
Multi-issue	2.048	(1.248)	2.441	(1.351)⁎	2.650	(1.393)⁎	2.317	(1.298)⁎
Other	– base level –							
Membership size	0.388	(0.285)			0.171	(0.159)		
United Nations			2.556	(1.429)^⁎^				
Text age	−0.307	(0.162)^⁎^	−0.349	(0.173)^⁎⁎^	−0.261	(0.154)^⁎^	−0.248	(0.143)^⁎^
Constant	−1.477	(1.461)	0.039	(1.222)	−1.850	(1.483)	−0.339	(0.827)
N	50		50		50		50	
AIC	69.257		67.606		67.626		67.186	

Notes: Dependent variable = normative commitment to EPI (binary variable); β = logit coefficient; S.E. = standard error; AIC = Akaike Information Criterion; Significance levels: ⁎ p< 0.10, ⁎⁎ p < 0.05; ⁎⁎⁎ p < 0.01.

## 2 Experimental Design, Materials and Methods

Table 1 gives an overview of the IGOs covered in this data article. The 78 IGOs selected represent a sample of the 347 IGOs originally covered by the Correlates of War (CoW) dataset [2]. It was necessary to reduce the number of IGOs in order to be able to manage the coding of the primary law texts. The IGOs selected comprise all energy-related IGOs (i.e., eight) and 28 environment-related IGOs. Regarding the latter, we sought to include the entirety of environment-related IGOs, but for some we experienced difficulties in getting access to the organizations’ primary law texts or the status of some organizations had changed since the compilation of the original CoW dataset.^1^ Besides IGOs working on energy and environmental issues, economy-related IGOs are important for assessing the commitment to EPI, CPI and/or EnPI. We drew a random sample of 27 organizations that represent bank/funds, commodity, and general economy-related IGOs. Furthermore, we randomly selected IGOs working on topics related to health, transportation, and science and education. Lastly, we drew a random sample of multi-issue organizations and IGOs that fall into the residual category ‘other’. For each of the categories health, transporation, science and education, multi-issue and ‘other’ we randomly selected three organizations. We relied on the study by Blake and Payton (2015) for assigning the IGOs to the different categories [3]. For energy-related IGOs, we followed Van de Graaf and Colgan (2016) [4].

Table 2 gives an overview of the statements on which we relied in order to code the individual IGOs’ normative commitment to policy integration. To this end, we used the dimensions ‘external’ and ‘internal’ integration as suggested by Biermann et al. (2009) [5]. The second set of dimensions refers to ‘coordination’ and ‘harmonization’ as put forth by Nilsson and Persson (2017) [6], Persson et al. (2018) [7] and Runhaar et al. (2014) [8]. The coding of the data took place in two steps. In a first step, three persons coded the data independently; two persons coded it a second time after having discussed cases where the assigned codes deviated.

Table 3 reproduces the summary statistics of the explanatory variables used in the research article by Tosun and Peters [1], but with two differences. While the analyses included in the research article rely on an indicator for assessing whether the *United Kingdom* is a member of an IGO, Table 3 reports the summary statistics for the membership of *France*. The second variable that deviates from Tosun and Peters is *Text age*, which is a scale ranging from 1 (shortest time elapsed) to 10 (longest time elapsed) for capturing the years elapsed since the adoption of the primary law text on which the coding draws (see Table 1). To produce this variable, we used the deciles of the number of years elapsed since the adoption of the original or amended primary law texts. The minimum number of years elapsed since the adoption of the relevant primary law text is zero years (Energy Community Treaty) and the maximum number 72 years (United Nations Food and Agriculture Organization).

Table 4 is a robustness check of the logit models estimated by Tosun and Peters [1], in which the variable *United Kingdom* is substituted by the variable *France*. In Table 5, the variable *Text age* replaces the variable *Post 1987*.

## Footnotes

1 We excluded the following IGOs due to missing data or changes in their legal status: Baltic Environmental Forum; Global Environment Facility; International Commission for the Hydrology of the Rhine Basin; International Pepper Community; International Board for Plant Genetic Resources; International Coral Reef Initiative; International Marine Reserve for the Western Mediterranean; Mediterranean Water Network; Multilateral Fund for the Implementation of the Montreal Protocol; North Atlantic Salmon Conservation Organization.
