# Supplementary material for: Dataset of statements on policy integration of selected intergovernmental organizations
Source: Data Brief. 2018 Feb 28;17:1391–404. doi: 10.1016/j.dib.2018.02.064 (PMC5854873; doi:10.1016/j.dib.2018.02.064)
Supplement: Supplementary file 1 — Supplementary material [file mmc1.docx]

Dear editor of Data in Brief,

I herewith declare that my co-author and I have no conflict of interest with this publication in Data in Brief.

Sincerely yours,

Jale Tosun
